# Demonstration of static electricity induced luminescence

**DOI:** 10.1038/s41598-022-12704-5

**Published:** 2022-06-02

**Authors:** Kazuya Kikunaga, Nao Terasaki

**Affiliations:** Sensing System Research Center, National Institute of Advanced Industrial Science and Technology, 807-1 Shuku-Machi, Tosu, Saga 841-0052 Japan

**Keywords:** Imaging and sensing, Electrical and electronic engineering

## Abstract

Can we visualise static electricity, which everyone in the world knows about? Since static electricity is generated by contact or peeling, it may be a source of malfunction of electronic components, whose importance is steadily increasing, and even cause explosion and fire. As static electricity is invisible, makeshift measures of static electricity are taken on various surfaces; there is also a common view that it is hard to take effective measures. Here we present a specific luminescent material, SrAl_2_O_4_: Eu^2+^, which emits light at excitation by an electrostatic charge in the air. Till now, in the interaction between electricity and luminescent materials, it was considered that emission of light is enabled by accelerated particles colliding with the luminescent material *in vacuo*. There have been no reports on luminescent materials being responsive to low-energy electrostatic charges under atmospheric pressure. Using SrAl_2_O_4_: Eu^2+^ luminescent material discovered by us, we succeeded for the first time in static electricity visualisation in the form of green light. In addition to the fact that such static electricity induced luminescence assists in solving electrostatic-related problems in the industry, it also provides a new measurement method that facilitates the observation of previously invisible electric charges in the air.

## Introduction

Static electricity is well known to most people, and everybody must have experienced sparks (that is, electrostatic discharge: ESD) when a charged person puts their fingers close to a metallic car or door. Just before one touches a metal, electrons are emitted from the metal due to the strong electric field formed between the charged finger and metal. This induces dielectric breakdown in the air, and this electrostatic discharge is accompanied by finger tweaking, light and sound. This type of electrostatic discharge is also called spark discharge, it emits light due to the ionisation of gas atoms in the air. However, as the emitted light intensity is extremely weak, it can hardly be seen in a well illuminated place. The potential of a charged body that induces electrostatic discharge is as high as several kV, but the average current is very weak, about 10^−5^ A. Such static electricity is reported to cause various failures and disasters in the industry^1,2^. Furthermore, electronic devices, e.g., large-scale integrated circuits, that have emerged due to advances in the semiconductor miniaturisation technology and electronic device systems equipped with them are very vulnerable to static electricity, which causes issues such as ESD damage and device failure.

Static electricity was discovered in ancient times, and around 600 BC, the Greek natural philosopher Thales described the attractive power of amber when it is rubbed with fur. In 1600, Gilbert found that apart from amber, various substances possess similar attractive force, and these substances were collectively named ‘electrica’. Cabeo discovered that the rubbed electrica also had the effect of repelling or attracting the material^3^. In 1733, du Fay discovered that charged objects of identical type repel each other, and charged objects of different type attract each other, assuming that there are two types of electricity, positive and negative^4^. In 1750, Franklin considered that every object contains an electric fluid in their respective portion, pointing out that its excess or deficiency is the ‘charged state’; this led to the discovery of polarity classified as positive (excessive) and negative (deficient)^5^. In 1836, Faraday demonstrated that the measurement of a charge depends on the observer’s electrical state and developed the Faraday cage to measure static electricity quantitatively for the first time. Thereafter, along with the Industrial Revolution, the research on electricity utilisation had intensified. Electrostatic-related issues in the industry were in focus since approximately 1970 that encouraged active research on measurement techniques for industrial applications. As a non-contact static electricity measurement technique, electrostatic voltmeters of various dielectric electrodes (rotating^6^ or standing^7^, vibrating^8^), field mill^9^, micro-electromechanical system sensors^10,11^, and methods using sound waves^12–14^ were developed. In addition, the electrostatic charge tends to localize in the plane, so it is important to grasp the planar two-dimensional distribution of charges. Therefore, to measure static electricity, methods for its screening point by point^15,16^, scanning probe microscopy^17–20^, and techniques using an array of sensors^21,22^ have been developed. Based on these methods, static electricity imaging on a computer screen became possible by combining the amount of static electricity and its spatial position. However, these techniques had a low spatial resolution and poor real-time performance. On the other hand, it is possible to increase the area and realize real-time performance by combining light with a camera, so static electricity measuring techniques using the electro-optic effect such as the Pockels effect^23,24^ and the Kerr effect^25,26^ have been developed. However, in a medium such as a crystal plate or gas, the Pockels and Kerr effects were weak, and the detection sensitivities were low. In this way, despite various challenges during 2700 years since the discovery of static electricity, it was not possible to clearly recognise static electricity with a human eye and to observe its behaviour.

In the meantime, luminescent materials have been used in different research fields as a tool for observing different physical phenomena. Examples include biomarkers or fluorescent materials incorporated in light sources and visualisation displays^27^, electroluminescent materials^28,29^, Long-persistent phosphors^30,31^, and further mechanoluminescent materials enabling visualisation of mechanical stress^32,33^. There are cathodoluminescence materials^34^ as well that emit light at the absorption of electrons accelerated *in vacuo*. However, an observation of static electricity in the air at atmospheric pressure and room temperature has not been reported using these types of materials. We have chosen a specific luminescent material, and emission of which is excited by charged ions generated in the air and succeeded in visualising static electricity.

## Results and discussion

### Charge generated by corona discharge in air, and SrAl_2_O_4_: Eu^2+^ light emission

**Figure 1:**
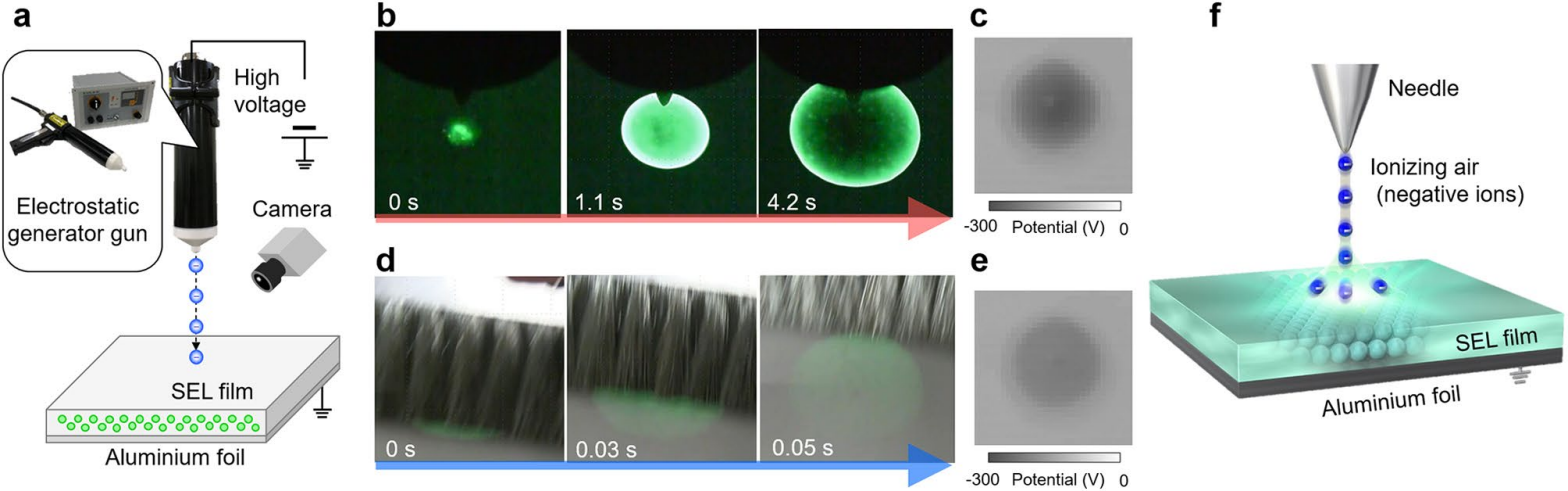
Static electricity induced luminescence (SEL) induced by charging and discharging. (**a**) Setup for measuring static electricity induced luminescence. (**b**) SEL at charging with electrostatic generator gun. (**c**) Surface potential distribution on the SEL film. (**d**) SEL at discharging by anti-static brush. (**e**) Surface potential distribution on SEL film after brushing. (**f**) Ion movement during charging.

Light emission peak around the wavelength of 510 nm was observed directly under the needle electrode in the device shown in Fig. 1a, immediately after subjecting static electricity induced luminescent film (SEL film: resistance ~ 10^14^ Ω) made of a mixture of SrAl_2_O_4_: Eu^2+^ and an acrylic resin to corona discharge by means of an electrostatic generator gun (Fig. 1b). Further, the initially observed light emission area expanded with time from the centre, its brightness saturated and whitened out even with a commercially available digital camera with time, while the light emission intensity decreased in the centre. The light emission decayed upon stopping the corona discharge. At this time, the heat of the sample was measured by thermography, and the temperature change in the light emitting area was 0.5 °C or less. As this light-emitting phenomenon, probably, relates to static electricity, it is called Static Electricity Induced Luminescence (SEL); and the substance that induces this phenomenon is called SEL material. The results of measuring the surface potential distribution of the SEL film are shown in Fig. 1c. The electrostatic charge formed by the corona discharge on the insulating SEL film is evidenced by the surface potential distribution, and the location of this charge coincided with the light emission area in Fig. 1b. In the case of dielectric breakdown in air, such as electrostatic discharge, spark discharge and short-circuit occur between the electrodes if the electric field strength exceeds approximately 3 MV/m at normal temperature and pressure^35^. As the applied electric field strength in this experiment was about 0.8 MV/m, and it is extremely inhomogeneous, there is no spark discharge, but corona discharge occurs in the region where electron avalanche concentrates in the vicinity of the electrode. The electrons near the needle electrode collide with the neutral air molecules under a strong electric field at the beginning of the discharge. The neutral molecules are ionized into positive ions and electrons, the positive ions move toward the negative electrode under the electric field, and the electrons continue to ionize other neutral molecules. The electrons will enter the plasma region after leaving the ionization region. The electric field in the plasma region cannot provide sufficient energy for the electrons. Therefore, the electrons combine with the neutral air molecules to form negative ions in the plasma region, then enter the drift region, and move towards the ground electrode^36^. In other words, in corona discharge, negative ions are emitted from the needle electrode and are incident to the SEL film owing to the potential difference between the needle electrode and the ground (Fig. 1f). The negative ions include OH^−^, NO_3_^−^, HCO_3_^−^^37,38^. Negative charges are formed when these negatively charged ions collide with the SEL film. This is the initial stage of the luminescence process demonstrated in Fig. 1b when it is supposed that the electric charge excites emission in the luminescent material due to radiative electronic transitions in Eu^2+^. Then, negative ions continuously generated by the corona discharge propagate alongside the SEL film as a negative potential is formed in the initially charged place. It is presumed that this process was observed as light emission expanding from the centre. Although this phenomenon differs in speed of the light emission area extension depending on the applied voltage in the range of 4–11 kV, similar results were obtained. This experiment was also tried with a positive electrode needle, and the same results as the negative electrode needle were obtained, and there was almost no difference between positive and negative.

Next, the charged SEL film was brushed with an anti-static brush. In the SEL area, light emission persisted right after the brushing as shown in Fig. 1d and Supplementary Video 1. Figure 1d shows the observation results in the bright field, and bright light emission was induced by the brushing. This anti-static brush is a self-discharge type destaticising tool for suppressing static electricity. The charge is removed through the thin conductive fibres at the tip of the anti-static brush, which provides grounding. Another, when the conductive fibre gets close to a charged object, a locally generated high electric field ionises the air layer between them, causing weak corona discharges. In Fig. 1d and Supplementary Video 1, it is considered that the charge transfer occurred due to the grounding and the corona discharge, and the phenomenon of the same light emission as Fig. 1b was observed. The maximum surface potential (Fig. 1c,e) of the SEL film before and after the brushing decreased from 285 to 160 V. In the surface potential measurements in this study, the distance between the sensor and the SEL film was 0.5 mm, the capacitance of a sensor of a 1 mm × 1 mm square was about 1.8 × 10^−14^ F; hence, a voltage of 1 V corresponded to 1.8 × 10^−14^ C mm^−2^ charge density. Consequently, the total electrostatic charge calculated from the results of surface potential distribution in Fig. 1c was 9.3 × 10^−10^ C. Also, the electrostatic charge was 6.8 × 10^−10^ C on the SEL film after the brushing. Luminescence was induced upon a charge difference of 2.5 × 10^−10^ C before and after brushing. This indicates that the SEL may have been induced with a very low-energy charge.

**Figure 2:**
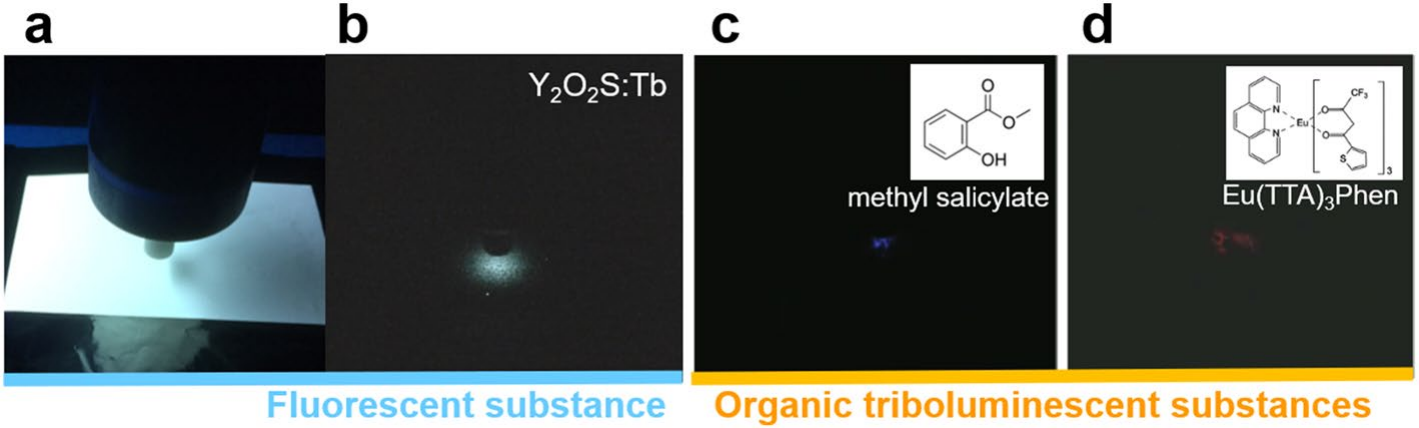
Light emission when luminescent substances was irradiated with corona discharge. (**a**) State of an experiment when the sample was irradiated with corona discharge. (**b**) White light observed in Y_2_O_2_S:Tb, which is a fluorescent substance. (**c**, **d**) Blue light and Red light observed in methyl salicylate and Eu(TTA)_3_Phen, which are an organic triboluminescent substances.

The phenomenon of light emission in response to electric charge is known as cathodoluminescence or electroluminescence. Generally, those light emission phenomena occur when electrons collide with a substance, such as a fluorescent material, but this requires the injection of high-energy electrons in an environment like a vacuum where there is no energy attenuation. For confirmation of the SEL phenomenon, similar experiments were conducted using other fluorescent substances (ZnS: Cu, Al, ZnS: Ag, Al, Y_2_O_2_S: Tb, Sakai Chemical Co., Ltd., Osaka, Japan) and organic triboluminescent substances applied in cathodoluminescence and electroluminescence, but no clearly recognisable light emission was observed (Fig. 2). The material used in this research may demonstrate luminescence (afterglow) excited by ultraviolet (UV) rays or blue light, heat or mechanical action. In Fig. 2b,c, it is considered that the light emission was excited by the UV rays accompanying the discharge. On the other hand, Fig. 1 shows a different emission compared to Fig. 2. If UV was generated by the irradiation of the corona discharge, SEL would be considered to emit light. Therefore, we placed an UV transmitting glass on the SEL film and the same experiment shown in Fig. 1 was carried out, but no light emission in SrAl_2_O_4_: Eu^2+^ was observed. In other words, the contribution of excitation by UV rays and blue light is small, indicating that it is highly possible that the ions contributed to SEL. Also, since the temperature change was small, it is considered that the contribution of excitation by heat is also small. Moreover, although triboluminescence can be considered in brushing, the contribution of typical triboluminescence is considered to be small because the luminescence phenomenon in the uncharged area could not be confirmed. Therefore, the light emission from the charged area caused by brushing with an anti-static brush cannot be explained by these phenomena, and the contribution of static electricity to the luminescence is possible.

**Figure 3:**
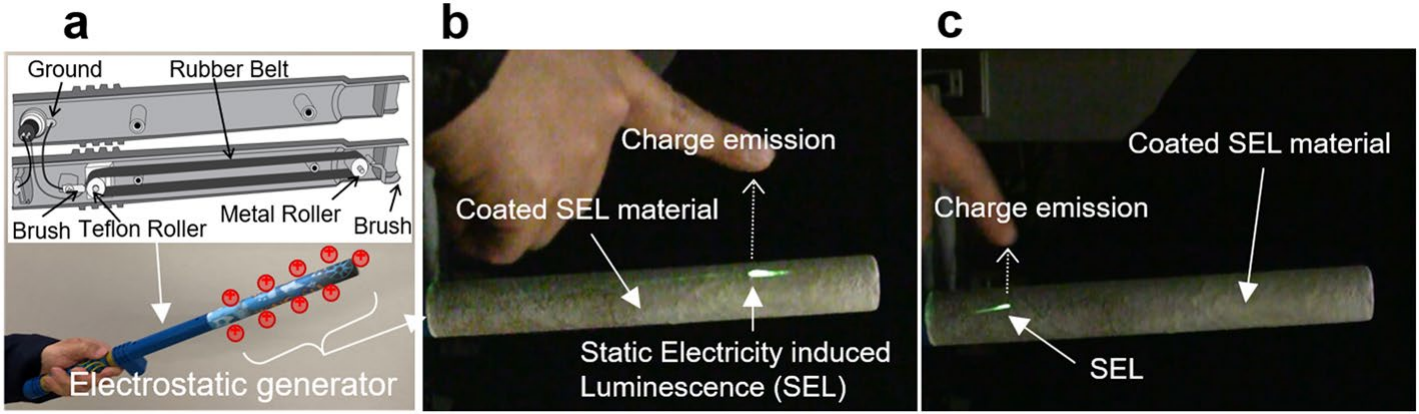
Static electricity induced luminescence (SEL) at places where charges were emitted from a Van de Graaff-type electrostatic generator to a finger. (**a**) Scheme of electrostatic charge generation. (**b**) SEL due to discharge between the finger and the electrostatic generator. (**c**) SEL when the finger is moved from left to right.

While we performed the experiments in which SEL was induced by the injection of electric charges, the same can be induced by electric charge release. For verification, a setup was prepared, in which a Van de Graaff-type electrostatic generator (Fun Fly Stick Magic Levitation Wand) that generates static electricity by friction and peeling was coated with a mixture of SrAl_2_O_4_: Eu^2+^ and a resin (Fig. 3). In the Van de Graaff-type electrostatic generator, metal and Teflon pulleys are connected by a rubber belt, and a cardboard pipe is connected to the tip. By rotating them with a motor, a high voltage of about 20–30 kV is generated due to repeated friction and charge separation. When approaching a finger to the electrostatic generator, the discharge proceeds in the place at the shortest distance between the finger and the electrostatic generator, and light is emitted as shown in Fig. 3b. When moving the finger, the light emission area shifts correspondingly (Fig. 3c and Supplementary Video 2). At this time, there was no temperature change in the light emitting area, which was the same as room temperature. This indicates that luminescence was observed when the charge was released from the electrostatic generator toward the finger. Thus, the SEL material manifests the phenomenon of luminescence during both charging and discharging processes.

### High potential SEL material SrAl_2_O_4_:Eu^2+^ possessing light emission in response to a low-energy charge

**Figure 4:**
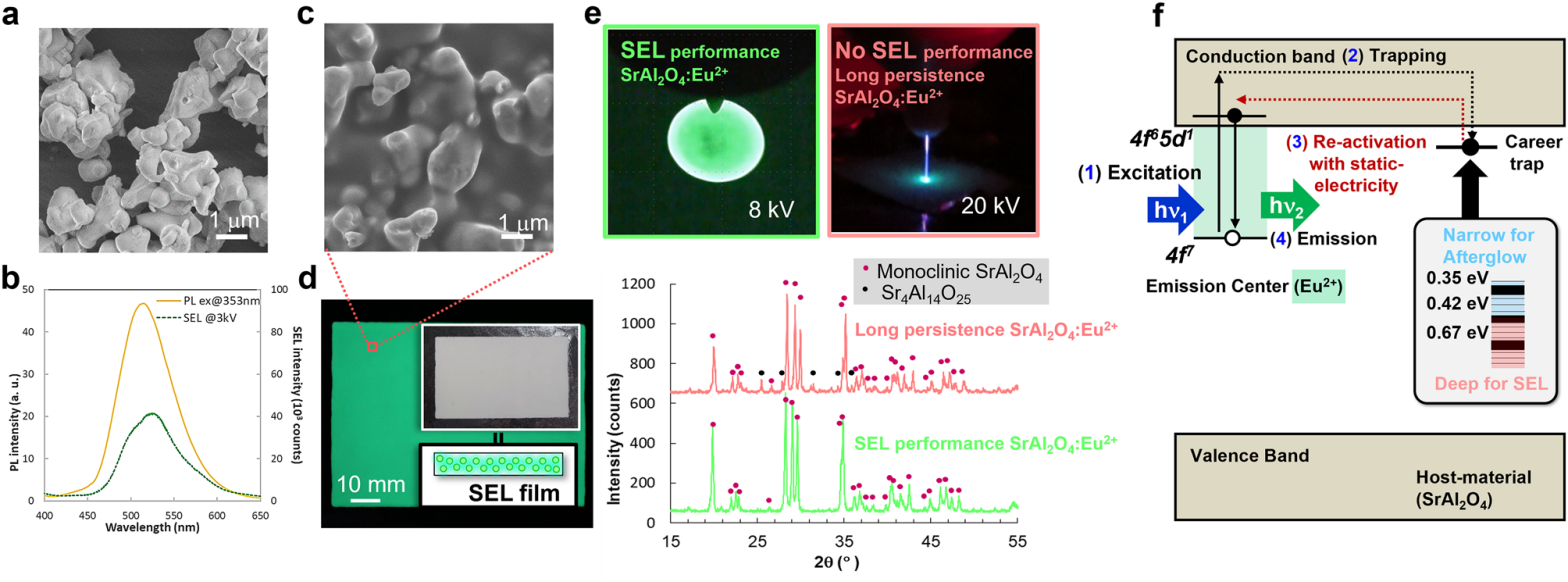
A SrAl_2_O_4_: Eu^2+^ static electricity induced luminescence material (SEL). (**a**) Scanning electron microscopy (SEM) of SEL material SrAl_2_O_4_:Eu^2+^. (**b**) Emission spectrum of SrAl_2_O_4_:Eu^2+^. (**c**, **d**) SEM and Film of SEL prepared by mixing SrAl_2_O_4_:Eu^2+^ and an acrylic resin. (**e**) X-ray diffraction of SEL performance and no SEL performance SrAl_2_O_4_:Eu^2+^. (**f**) The expected luminescence mechanism of SEL.

**Figure 5:**
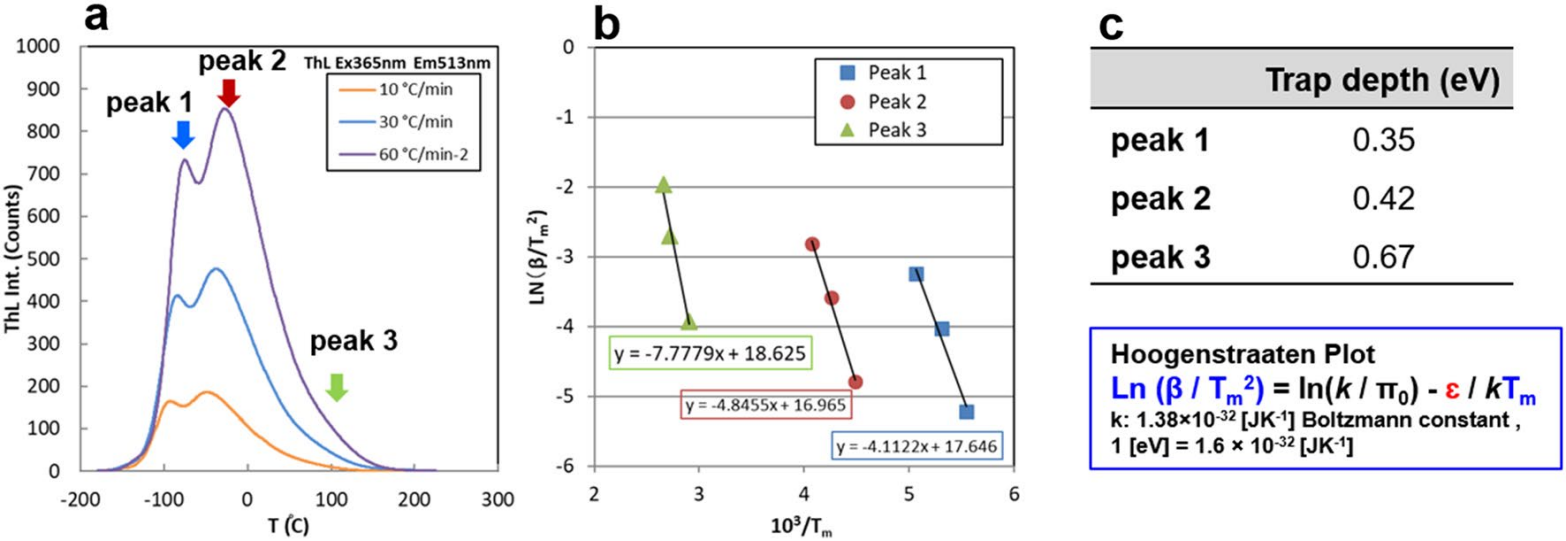
Thermoluminescence (TL) measurement of SrAl_2_O_4_: Eu^2+^ as a SEL material. (**a**) TL curves measured at different heating rates. (**b**) Hoogenstraaten plots and **c**, Calculated trap depths.

To obtain an SEL material possessing bright light emission in response to a low-energy charge, it is necessary to select a smart material taking into account not only the chemical composition but also the crystal axis and other features. The experiment described above was performed using SrAl_2_O_4_: Eu^2+^, which is also known as a luminescent, phosphor, and triboluminescent material. Figure 4a–f show the scanning electron microscopy (SEM) image, emission spectrum, general view of the SEL film, x-ray diffraction (XRD) and the presumed mechanism of SEL in SrAl_2_O_4_: Eu^2+^. The XRD spectrum indicates a pure monoclinic SrAl_2_O_4_ structure, where AlO_4_ tetrahedra share oxygen atoms to form a three-dimensional network. As described in previous reports on SrAl_2_O_4_: Eu^2+^, phosphors and triboluminescent materials with Sr^2+^ inserted for charge compensation impart a slight distortion to the alumina tetrahedra. Divalent Eu^2+^ was dopped in the Sr^2+^ site and serves as a luminescence centre. Because the SEL spectrum comprises a broad peak centred at 520 nm and is similar to the photoluminescence (PL) spectrum, both are obviously related to the light emission caused by 4f^7^–4f^6^5d^1^ transitions in divalent Eu^2+^ ^39^. In the PL spectrum, afterglow spectrum, and SEL spectrum, no sharp emission peak near 630 nm derived from Eu^3+^ was observed. Further, based on the thermoluminescence curves (Fig. 5a–c), carrier traps can be distinguished with the depths of 0.67, 0.42 and 0.35 eV contributed to the light emission. Therefore, the following mechanism can be presumed. In the SEL material, ultraviolet to blue light is absorbed to excite the carriers, where part of them promptly diffuse to the Eu^2+^ luminescence centre resulting in light emission, but some carriers are captured by traps. After the excitation by UV to blue light, afterglow from the thermal excitation of carriers in shallow traps is observed from the SEL film. It is supposed that when an electric charge is injected into the crystal, the captured carriers release and recombine with holes at Eu^2+^, leading to the observed light emission as so-called SEL. Also, the light emission disappears after SEL, and it is observed as the spread of dark spots that follow the spread of SEL from the center region as shown in Fig. 1b. This suggests that the injected charge affects the trapped carriers as shown in Fig. 4f^40^. In Fig. 1, the negative ions that reach the SrAl_2_O_4_: Eu^2+^ surface, can transfer electrons to SrAl_2_O_4_: Eu^2+^. This is also the case with positive ions, the positive ions can receive electrons from SrAl_2_O_4_: Eu^2+^. At the moment, the interaction of positive and negative charges with respect to SEL is unknown, but we assume that charges or ions act as external stimuli, and SEL would be a mechanism similar to long-persistent phosphors and mechanoluminescent materials. The mechanisms of direct light emission by static electricity on the SEL performance should continue to study, but the obtained light emission is strong enough to visualise static electricity. Furthermore, the SEL offers promising perspectives; including the improvement of luminous efficiency, as well as possible multicolour emission by controlling the crystal field and carrier trap levels by adjusting the material composition. In addition, regarding the SEL efficiency in luminescent materials, emission intensity may depend on crystal orientation (Fig. 4e). This is that the SEL reported in this study is completely different from previously known phenomena and indicate that the contribution of excitation by static electricity can be controlled by selecting a smart material and adopting a suitable material design.

### Visualisation of static electricity by light emission

**Figure 6:**
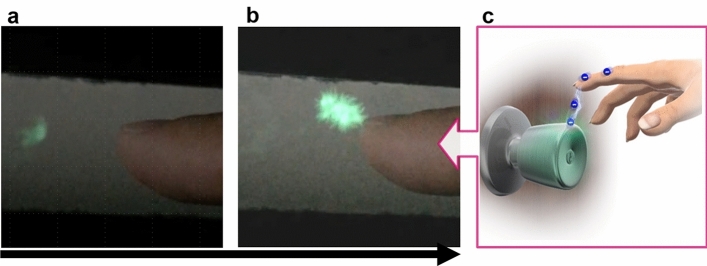
Static electricity induced luminescence (**a**, **b**) Before and just as a ‘spark’ is observed by electrostatic discharge through a charged finger. (**c**) Image illustrating electrostatic discharge generated by the doorknob.

Finally, Fig. 6 shows the SEL that occurs when a charged human finger is brought close to an aluminium foil coated with the SEL material. This time, the charged voltage of the person is approximately 3–4 kV, which was measured with a Body Voltage Meter. Figure 6a demonstrates the SEL observed when the distance between the finger and the conductor is 1–2 cm, and Fig. 6b demonstrates SEL following the spark caused by electrostatic discharge when the finger is brought closer to the conductor. Figure 6c is image illustrating electrostatic discharge generated by the doorknob, Fig. 6b and c show similar situations. In Fig. 6b, it is considered that a strong electric field was formed between the finger and the conductor, and spark discharge occurred because the Paschen’s law conditions were satisfied, so that SEL was induced by the generated ions. On the other hand, the light emission phenomenon was observed for at least 2 s before the occurrence of electrostatic discharge spark (Fig. 6a and Supplementary Video 3). There are three discharge regimes for DC low-pressure discharge in gas: dark discharge, glow discharge, and arc. The 'dark discharge' regime is characterized by a positive impedance in which voltage rises roughly linearly with current while producing little^41^. Since no visible discharge was confirmed in Fig. 6a, the SEL observed before this electrostatic discharge is also expected to occur in the dark discharge region (current ~ 10^−10^–10^−5^ A). In other words, Fig. 6 shows that the phenomenon in which ions were emitted from a charged human finger and entered to the SEL material was visualized in real time. The SEL would be obviously induced by a very low-energy charge. In the future, by advancing our knowledge about this phenomenon, we may be able to observe and discover unexplored new phenomena.

To summarise, in this research, we focused on static electricity and luminescent materials for its registration and succeeded for the first time in visualising the electric charge caused by static electricity. The light-emitting particles are the smallest electrostatic sensors existing in the world that emit light when charges are injected and output without requiring a power source. By applying the micro static electricity sensor, the static electricity of a three-dimensional object can be measured remotely in real-time with a camera; thus, SEL may be considered as an innovative static electricity sensing technique. Furthermore, SEL will open up new research areas enabling easy recognition and observation of so far invisible charges and may help in solving electrostatic problems in the industry.

## Methods

### Material

SrAl_2_O_4_: Eu^2+^ was synthesised using the solid-phase reaction method^32^. Stoichiometric amounts of α-Al_2_O_3_, Sr_2_O_3_ and Eu_2_O_3_ powders (Kojundo Chemical Lab. Co., Ltd, Saitama, Japan) were ground to form a homogeneous fine powder. The mixed powder was then pre-calcined in the air at 800 °C for 2 h, sintered in a reducing atmosphere at 1400 °C for 6 h and then ground into a fine powder 1–2 µm size microparticles (Fig. 4a).

### Characterisation methods

The phase, purity and crystal structure of the obtained material were determined by XRD (RINT-2000, Rigaku Co., Tokyo, Japan). A spectrophotofluorometer (FP6600, JASCO Co., Tokyo, Japan) was used for PL measurement performed at room temperature. Using SEM (Analytical thermal field emission SEM, JSM-7001F, JEOL. Co., Tokyo, Japan), particle size, the elemental composition in the microscopic region, as well as luminescence properties were evaluated.

### Film fabrication

A mixture of SrAl_2_O_4_: Eu^2+^ and photocurable acrylic resin (weight ratio: 3:7) was applied to the surface of an aluminium foil and cured with UV irradiation (0.5 mW/cm^2^, 30 min.) to prepare an SEL sheet (area: 50 mm × 50 mm, film thickness: about 50 µm).

### Corona discharge generation

An electrostatic generator gun (GC90, Green techno Co., Ltd., kanagawa, Japan) generated a corona discharge for 5 s under the condition of applied voltage: − 8 kV, current: 0.8 µA, diameter of a needle electrode: 5 mm, needle electrode-ground distance: 10 mm (Fig. 1a).

### Optical measurements of SEL

In the air, at a temperature of 20 °C and humidity of 30%, the SEL sheet was excited by UV irradiation (wavelength: 365 nm) for 60 s, and a voltage was applied 30 s later to generate corona discharge. The SEL was measured using a Commercial video camera (Canon ivis HF, Canon Inc., Tokyo, Japan) (Fig. 1).

### Surface potential measurements

Surface potential distribution were measured with an area of 30 × 30 mm^2^ and a spatial resolution of 1 mm using the device described in reference^22^.

## Supplementary Information

Supplementary Information 1.

Supplementary Video 1.

Supplementary Video 2.

Supplementary Video 3.

## Data Availability

The datasets generated during and/or analysed during the current study are available from the corresponding author on reasonable request.
