# Supplementary material for: Demonstration of static electricity induced luminescence
Source: Sci Rep. 2022 Jun 2;12:8524. doi: 10.1038/s41598-022-12704-5 (PMC9163190; doi:10.1038/s41598-022-12704-5)
Supplement: Supplementary file 1 — Supplementary Information 1. [file 41598_2022_12704_MOESM1_ESM.docx]

**Supplementary Information**

**Demonstration of Static Electricity Induced Luminescence**

Kazuya Kikunaga*, and Nao Terasaki

Sensing System Research Center, National Institute of Advanced Industrial Science and Technology, 807-1 Shuku-Machi, Tosu, Saga 841-0052, Japan

*E-mail :k-kikunaga@aist.go.jp

**Supplementary Videos**

**Description of Additional Supplementary Files**

**Supplementary Video 1.**

**Description:** Movie of static electricity induced luminescence at the discharging process by anti-static brush, illustrated in Fig. 1.

**Supplementary Video 2.**

**Description:** Light emission area shifts correspondingly when moving the finger.

**Supplementary Video 3.**

**Description:** Demonstration of static electricity induced luminescence filmed at the time of spark created by electrostatic discharge through a finger, described in Fig. 6.
